# Ethical and legal issues in mitochondrial transfer

**DOI:** 10.15252/emmm.201606281

**Published:** 2016-04-21

**Authors:** Ainsley J Newson, Stephen Wilkinson, Anthony Wrigley

**Affiliations:** ^1^ Centre for Values, Ethics and the Law in MedicineSchool of Public HealthUniversity of SydneySydneyNSWAustralia; ^2^ Department of Politics, Philosophy and ReligionLancaster UniversityLancasterUK; ^3^ Centre for Professional EthicsSchool of LawKeele UniversityKeeleUK

**Keywords:** Genetics, Gene Therapy & Genetic Disease, Metabolism

## Abstract

The US National Academies of Science, Engineering and Medicine recently provided conditional endorsement for mitochondrial transfer. While its approach is more conservative in some respects than that of the United Kingdom (which passed its own regulations in 2015), it marks a significant policy development for a potentially large implementer of this emerging intervention. In this perspective, we consider some of the ethical and legal aspects of these policy responses.

## Ethical and legal issues in mitochondrial transfer

Mitochondrial transfer (MT) techniques are being developed as one method of enabling at‐risk couples to avoid having a child with mitochondrial disease (Richardson *et al*, 2015). These conditions can affect multiple organ systems, are often debilitating and life‐shortening and at present cannot be cured.

Two main MT techniques are proposed: maternal spindle transfer (MST) and pronuclear transfer (PNT) (Richardson *et al*, 2015). MST is undertaken in oocytes and involves removing the spindle from an oocyte with affected mitochondria and inserting it into an enucleated donor oocyte, which then undergoes *in vitro* fertilisation. PNT involves creating an embryo using the intended parents' gametes and removing the pronuclei. This is then transferred into an early embryo created using the intended father's sperm and a donor oocyte and allowed to develop.

MT raises conceptual, ethical and legal issues (Nuffield Council on Bioethics, 2012). As well as the fundamental question of whether it should be allowed at all, these technologies raise more specific questions. Should they be categorised as a germline genetic modification? Should only male embryos be implanted? Do the children created have a right to know who the oocyte donor was? And what legal liabilities would and should ensue? Each of these is discussed below.

## What's in a name?

Nomenclature in relation to emerging biotechnologies often occurs haphazardly, or is bestowed by those who may not be key to its development. This is unfortunate, as the connotations of a particular term may adversely affect subsequent ethical, public and policy debates (Ravitsky *et al*, 2015).

Interventions to alter mitochondria in embryos are beset by multiple names, most of which have misleading connotations. To date, the MT technologies described above have been termed “mitochondrial donation”, “mitochondrial replacement”, “mitochondrial therapy”, “mitochondrial transfer” and “three‐parent IVF”. Here, we use “mitochondrial transfer” as the most accurate and normatively “neutral” term, albeit recognising that this is an imperfect descriptor given some aspects of MT technologies.

## Regulatory activity and policy development

The United Kingdom (UK) was the first country actively to regulate MT; passing regulations that came into force on October 31, 2015 (The Human Fertilisation and Embryology (Mitochondrial Donation) Regulations 2015 No. 572). The UK regulations allow clinical use of MT under licence. No licence applications have yet been received, although one is expected soon.

An important policy development also occurred in the United States in February 2016 when a specially constituted committee of the National Academies of Science, Engineering and Medicine (NASEM) sanctioned a slightly narrower use of MT than that regulated in the UK. NASEM recommended that “mitochondrial replacement techniques” should be considered for clinical implementation, subject to certain conditions, including that only male embryos should be implanted (NASEM, 2016).

These regulatory developments should also be considered in the light of the distinct approaches to regulation of reproductive technologies in these jurisdictions (Ouellette *et al*, 2005). The United Kingdom tightly regulates clinics providing assisted reproductive services and novel reproductive technologies. The United States, in contrast, takes a more laissez‐faire approach, which some claim is due to certain constitutional rights. This relative lack of regulation may be one reason why the NASEM report provides detailed recommendations as to the clinical implementation of MT.

## Genetically modified children? Ethical and legal implications

MT raises ethical and conceptual concerns over whether it is a form of germline gene therapy, and whether children born following MT are genetically modified. Unlike somatic therapies, germline modifications are not usually permitted due to the perceived risks for future generations. In response, we may ask both how MT should be classified and whether modifying the germline is always wrong.

There is as yet no consensus as to whether MT is a genetic modification or germline gene therapy, although key distinctions have been emphasised. First, germline therapies usually target nuclear DNA, whereas MT targets whole mitochondria outside the nucleus. Second, the transplanted mitochondria will only be heritable matrilineally and therefore will not be passed onto male offspring. This can be termed the “quasi‐heritability” of MT.

This lack of agreement over MT's status can also be observed in policy. Both UK policymakers and the NASEM report accept that MT technology is a form of heritable modification. But while key UK policy reports accept that MT has germline implications, they reject MT as a “genetic modification”—a unique policy turn—on the grounds that their working definition of what constitutes a “genetic modification” involves the heritable modification of only *nuclear* DNA (Public Health Directorate, 2014).

The NASEM report, in contrast, distinguishes between “genetic modification” and “germline modification” (NASEM, 2016, sect 3). NASEM considers genetic modification to be “changes to the genetic material within a cell” and germline modification to be “human inheritable genetic modification”. This distinction allows them to claim that: “MRT [MT] involves genetic modification, but that it constitutes…germline modification… only if used to produce female offspring” (NASEM, 2016, sect 3, p. 8).

Whether MT is a form of genetic modification depends both on how we define “genetic modification” and on the attributes of the specific MT technology employed. While MT would constitute a form of genetic modification under almost every current definition, whether it can always be classified as a germline gene therapy or whether it is classifiable as some other form of intervention is both a matter of recent debate and something that may have subsequent ethical implications for its use (Wrigley *et al*, 2015).

## Sex selection

One notable point of divergence between the British and American policy approaches is that while the NASEM Report recommends “restricting initial first‐in‐human clinical investigations to male embryos” (NASEM, 2016, sect 4, p. 6), the UK has rejected such an approach.

NASEM's main argument for this restriction is that, because mitochondrial inheritance is matrilineal, using only male embryos stops MT from being a “germline” genetic modification with its attendant (but theoretical) risks to future generations. The UK Human Fertilisation and Embryology Authority (HFEA), however, argued against a “male embryos only” policy on the grounds that “using sex selection after mitochondrial replacement would expose the embryos to additional intervention”, which might generate extra risk (HFEA, 2013, s.6.19). The UK Government also accepted the safety of *all* MT, regardless of the biological sex of the children who may be born.

The difference between NASEM and HFEA is therefore less about the ethics of sex selection *per se* than about weighing different kinds of risk. The HFEA has chosen to prioritise avoiding perceived short‐term risks to the first generation of children created (citing concerns about the extra biopsy needed for sex selection), whereas NASEM places greater emphasis on what it sees as longer‐term risks to the “gene pool”.

Another risk that should be countenanced—mentioned by neither the HFEA nor NASEM—is the risk of IVF failure (and thus more invasive interventions for women participating in MT) if the pool of “suitable” embryos is reduced by half, to male embryos only.

## Donor anonymity

Many jurisdictions give the genetic offspring of egg and sperm donors a right to identifying information about those donors. MT then raises the question of whether children born from its use should have similar rights. This has sometimes been couched in terms of whether egg donors for MT are more like “regular” gamete donors, or blood or organ donors. The HFEA, for example, has advised that “mitochondria donors should have a similar status to that of tissue donors” (HFEA, 2013, s.6.64).

Some of the supposed differences between mitochondrial egg donors and “regular” egg donors—which may justify *not* giving MT‐conceived children a right to know—are as follows. First, in “regular” gamete donation, the offspring are quite likely to resemble the donor in noticeable ways; this is not the case for mitochondrial transfer. Second, nuclear DNA is said to be constitutive of a person's genetic identity in ways that mitochondrial DNA (mtDNA) is not. In addition, “the child's sense of self would be inherited from their [social] parents” (HFEA, 2013, s.6.49).

Third, it might be argued that non‐disclosure of origins in “regular” gamete donation is more likely to involve serious deception than for mitochondrial transfer. In MT, the child will often be raised by its two main genetic parents; so if the child is not fully informed about its MT origins, it merely fails to know about an *extra* person's contribution. However, when “regular” donor‐conceived individuals are not informed, this may involve “passing off” one of its social parents as a genetic parent.

Against this, however, it may be argued that the role mtDNA plays in determining our physical development is uncertain and that there may therefore be a precautionary case for granting a right to know: *just in case* it turns out that mtDNA has a greater role biologically than we thought; or *just in case* it turns out that MT‐conceived children have a strong desire to know who their donor was.

One further reason to allow a right to know is that anonymous donation overlooks the role of oocyte donors (Haimes & Taylor, 2015). The process of donation and the risks that ensue is the same for MT as it is for “regular” donation. Rendering donation anonymous could therefore be said to undervalue this contribution.

## Legal risks?

While a comprehensive comparison of global legal regulation for MT is beyond the scope of this article, determining sound legal responses to MT and its possible harms remain live issues.

MT's potential risks may cause us to wonder about any liability for those who provide it. There are, for example, ongoing tensions in the literature around interactions between mitochondrial and nuclear DNA, and “contamination” by residual mitochondria (Hamilton, 2015).

If (and it is a “big if”) these risks transpire, might this lead to claims for compensation against those providing the technology? Could a child born with sub‐optimal health as a result of MT successfully bring an action against those who provided the treatment? The answer, in many jurisdictions, is “unlikely” for three main reasons.

First, in countries with common law legal systems, most claims like this—so‐called wrongful life claims—tend to fail because of difficulties in making the particular circumstances fit the elements of a cause of action in negligence. This includes the need for the person to show that they have been so badly harmed that they would be better off not having been born. The standard of care for MT is also not yet established, as it is experimental. An action in negligence has little to do with a particular jurisdiction's regulatory approach; rather, it involves (amongst other things) comparing the delivery of a treatment against an established standard.

Second, some types of MT might actually lead to a different person being born altogether; such that no harm is done to a particular person (Wrigley *et al*, 2015). This issue arises from philosophical reflection upon the nature of harms and wrongful life claims. For an individual to claim to have been harmed by coming into existence, they would have to show that they were worse off as a result of MT. But with some MT techniques, the intervention happens prior to fertilisation. If MT had not taken place, an entirely different person would have been born. Thus, the child claiming harm from MT would not have existed and cannot claim to have been harmed by its use.

Third, the experimental nature of this intervention differentiates MT from many other wrongful life decisions. Any legal claim would presumably not be about a test being done incorrectly, or a diagnosis missed. Instead, it is the technology itself that may be risky or imperfect no matter how well it is applied. If a couple has received appropriate counselling as to these risks and is still prepared to go ahead, then the resulting child would be unlikely to have an action against the treating health professional.

## Ethics and the future of human reproduction

Mitochondrial transfer raises a challenging range of ethical and regulatory questions and, as it is rolled out into clinical practice, more are sure to emerge. It also encourages us to revisit more familiar ideas such as “germline genetic modification”, to ask whether such modifications are *always* wrong, and to consider whether the concept itself is “fit for purpose”. MT similarly makes us think anew about the basis on which donor‐conceived people are given a right to know their biological origins and about who else should have this right. While some such issues are new, many of them are not unique to MT and similar questions are already being asked about other developments in human reproduction, such as *in vitro* generated (“artificial”) gametes and uterus transplants (Newson & Smajdor, 2005; Catsanos *et al*, 2013; Wilkinson & Williams, 2015). Our ethical and regulatory responses to emerging reproductive technologies need to deal with a wider range of issues than ever before.

## Conflict of interest

The authors declare that they have no conflict of interest.
